# Akneiformer Mogamulizumab‐assoziierter Rash

**DOI:** 10.1111/ddg.15816_g

**Published:** 2025-11-14

**Authors:** Inga Hansen‐Abeck, Glenn Geidel, Finn Abeck, Anne Menz, Stefan W. Schneider, Nina Booken

**Affiliations:** ^1^ Klinik und Poliklinik für Dermatologie und Venerologie Universitätsklinikum Hamburg‐Eppendorf, Hamburg; ^2^ Institut für Pathologie Universitätsklinikum Hamburg‐Eppendorf, Hamburg

**Keywords:** Mogamulizumab, Mogamulizumab‐assoziierter Rash, Mycosis fungoides, primär kutane T‐Zell‐Lymphome, Sézary‐Syndrom, cutaneous t‐cell lymphoma, mogamulizumab, mogamulizumab‐associated rash, mycosis fungoides

Sehr geehrte Herausgeber,

Der Mogamulizumab‐assoziierte Rash (MAR) ist eine der häufigsten Nebenwirkungen des gegen den CC‐Chemokinrezeptor‐4 (CCR4) gerichteten Antikörpers Mogamulizumab, der zur Zweitlinientherapie gegen die Mycosis fungoides (MF) und das Sézary‐Syndrom (SS) zugelassen ist.^1^ In der Zulassungsstudie (MAVORIC) wurde die Häufigkeit des MAR mit 24 % angegeben. Er war die häufigste Nebenwirkung, die zum Therapieabbruch führte.^1^

Es wurden verschiedene Ausprägungen des MAR beschrieben, wobei sich vier klinisch vorherrschende Präsentationsformen unterscheiden lassen: *(1)* follikulotrope, Mycosis‐fungoides‐ähnliche Plaques der Kopfhaut mit Alopezie, *(2)* Papeln und/oder Plaques, *(3)* photoakzentuierte Dermatitis sowie *(4)* morbilliforme oder erythroderme Dermatitis.^2^, ^3^ Darüber hinaus wurden auch Einzelfälle seltener Varianten publiziert, wie die palmoplantare Hyperkeratose oder die Imitation eines Lupus miliaris disseminatus faciei.^3^, ^4^, ^5^, ^6^ Hinsichtlich des histologischen Reaktionsmusters wurden drei Hauptformen des MAR identifiziert, wobei Mischformen häufig sind: *(1)* spongiotische/psoriasiforme Dermatitis, *(2)* Interface‐/lichenoide Dermatitis und *(3)* granulomatöse Dermatitis.^3^, ^7^

Wir berichten über einen 42‐jährigen männlichen Patienten mit follikulotroper MF im Stadium IIB (T3 N0 M0 B0) mit großzelliger Transformation. Nach Einleitung einer Therapie mit Bexaroten begannen wir aufgrund eines Krankheitsprogresses (*modified Severity Assessment Tool* [mSWAT] 56) die Behandlung mit Mogamulizumab, welches nach Prämedikation mit Clemastin und Paracetamol verabreicht wurde. Weitere Medikamente, insbesondere Glukokortikosteroide oder Antibiotika, wurden nicht eingenommen. Nach fünf Zyklen Mogamulizumab (1 mg/kg Körpergewicht) berichtete der Patient über das Auftreten neuer Hautveränderungen, während sich die Tumorknoten und ‐plaques deutlich zurückgebildet hatten (Abbildung 1a–c). Klinisch zeigten sich follikulär betonte Pusteln im Gesicht, am Rumpf, an Armen und Beinen, passend zu einer akneiformen Dermatitis. Es bestand kein makulopapulöses Exanthem, keine Blasenbildung oder Epidermolyse, sodass Arzneimittelreaktionen wie ein DRESS‐Syndrom oder eine toxisch‐epidermale Nekrolyse klinisch ausgeschlossen werden konnten. Histologisch zeigte sich eine pustulöse Follikulitis und Perifollikulitis mit perivaskulären lymphozytären Infiltraten und einem hohen Anteil an CD8‐positiven Zellen. Die akute, entzündliche und histiozytenreiche Reaktion mit starker CD8‐positiver Komponente sowie das polyklonale T‐Zell‐Rezeptor‐*Rearrangement* entsprachen dem histologischen Bild eines MAR (Abbildung 2). Aufgrund des multilokulären Auftretens und der klinischen Präsentation mit follikulären Pusteln passten die Hautveränderungen nicht zu den bisher beschriebenen Typen des MAR, sodass wir erstmals die Diagnose eines akneiformen MAR stellten. Wir begannen eine Therapie mit topischen Glukokortikosteroiden sowie topischen Antibiotika und führten die Behandlung mit Mogamulizumab fort. Da keine Besserung des MAR festzustellen war, erfolgte zusätzlich eine interne Behandlung mit Doxycyclin 200 mg täglich. Zwei Wochen nach Beginn der Doxycyclin‐Therapie berichtete der Patient eine deutliche Reduktion der Hautveränderungen und des Juckreizes. Ein Auslassversuch von Doxycyclin führte bereits nach kurzer Zeit zum Wiederaufflammen des MAR. Daher wurde Doxycyclin fortgeführt, ebenso wie die Therapie mit Mogamulizumab. Die MF zeigt sich nach zehn Zyklen Mogamulizumab weiterhin mit einem mSWAT von 16 kontrolliert (Abbildung 1e–f). Weitere Mogamulizumab‐assoziierte Nebenwirkungen traten bei dem Patienten nicht auf.

**ABBILDUNG 1:**
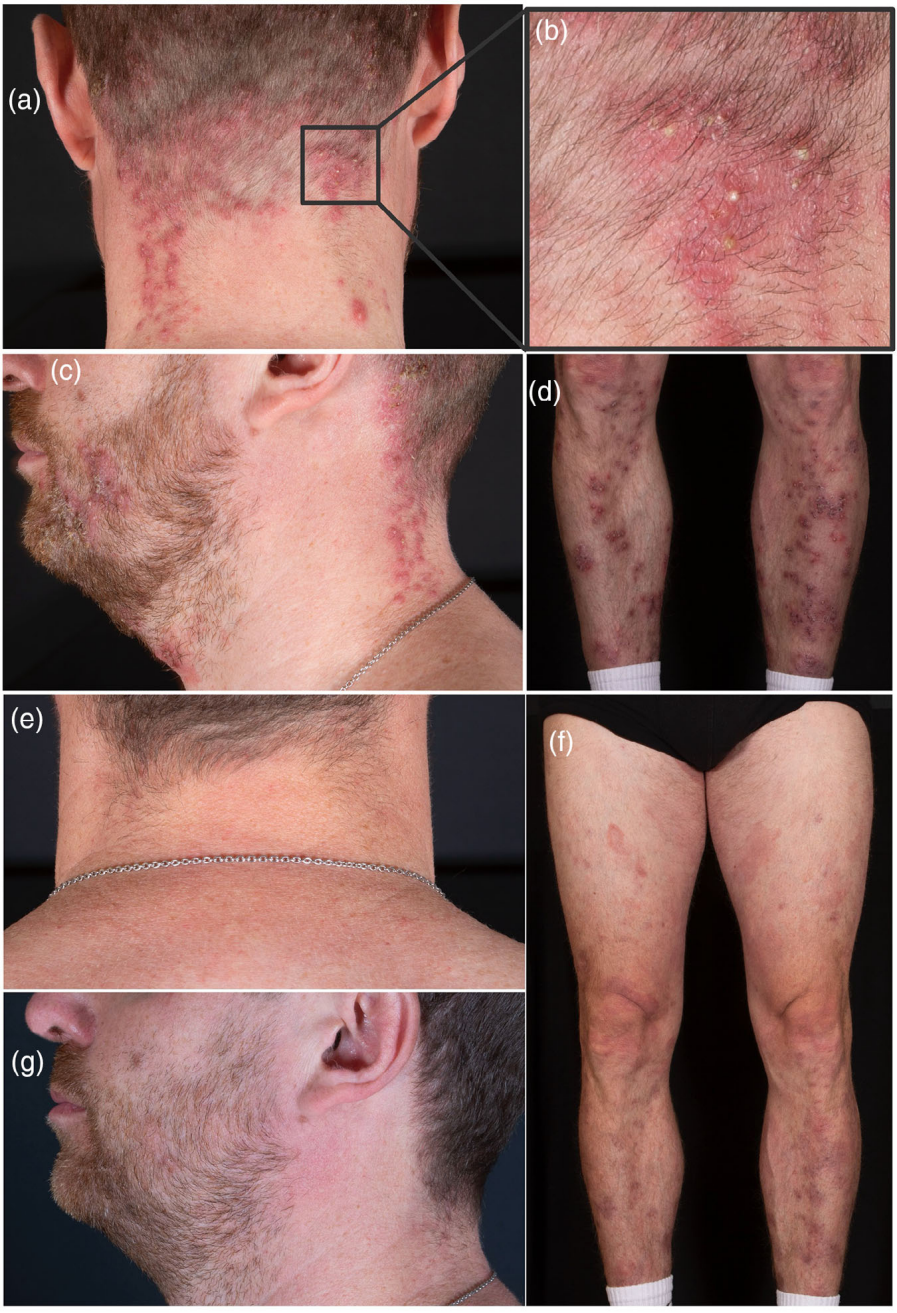
Akneiformer, Mogamulizumab‐assoziierter Ausschlag mit erythematösen Papeln und Pusteln am (a) Hals, (b) in der Vergrößerung, (c) an den Beinen und (d) im Gesicht. Darstellung nach 8‐wöchiger Behandlung mit Doxycyclin am (e) Hals, (f) an den Beinen und (g) im Gesicht.

**ABBILDUNG 2:**
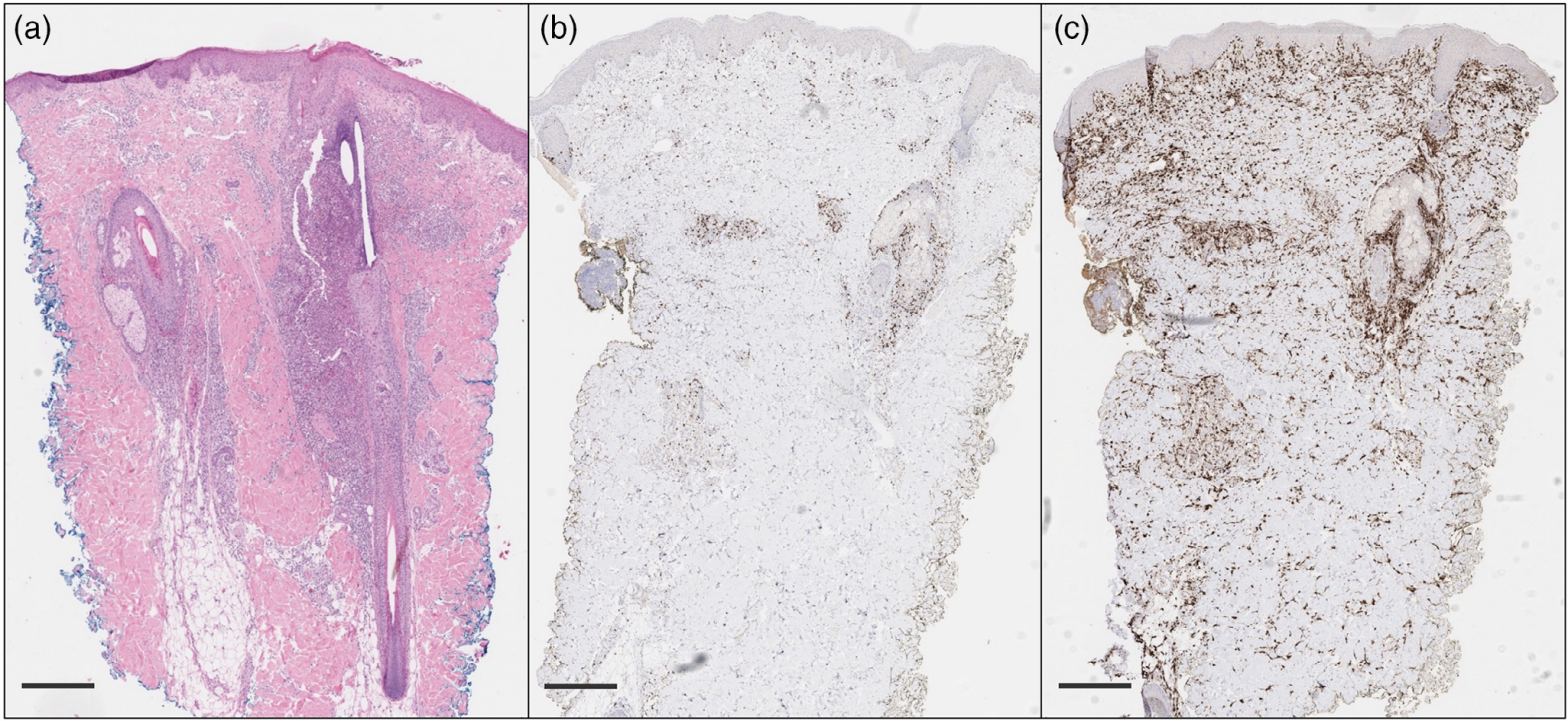
Histologisch zeigt sich eine neutrophilen‐ und histiozytenreiche Entzündung der Haarfollikel mit einem hohen Anteil an CD8⁺‐Zellen, vereinbar mit einem Mogamulizumab‐assoziierten Ausschlag. Maßstabsbalken: 500 µm. (a) Hämatoxylin‐Eosin‐Färbung mit akuter Follikulitis und Perifollikulitis, perivaskulären lymphozytären Infiltraten sowie neutrophilen‐ und histiozytenreicher Entzündung der Haarfollikel. (b) Immunhistochemisch imponiert eine ausgeprägte CD8⁺‐Komponente mit einem CD4‐zu‐CD8‐Verhältnis von 2:1. (c) CD163‐Färbung mit deutlichem Anteil histiozytärer Zellen.

Nach unserem Kenntnisstand handelt es sich um den ersten veröffentlichten Bericht eines akneiformen MAR. Bisher ist lediglich ein Fall eines MAR mit Pustelbildung beschrieben, bei dem ein pustulöser Ausschlag der Kopfhaut auftrat und aufgrund der Histologie als eosinophile Follikulitis diagnostiziert wurde.^8^ Ein akneiformes Exanthem ist eine häufige Nebenwirkung der Behandlung mit EGFR‐Inhibitoren.^9^ Die genaue Pathophysiologie des MAR ist noch nicht vollständig verstanden. Eine mögliche Erklärung könnte der Rückgang von CCR4‐exprimierenden regulatorischen T‐Zellen sein, was zur Aktivierung zytotoxischer CD8^+^ T‐Lymphozyten führt.^10^ Es sind Fallberichte mit follikulärer Beteiligung von Lymphozyten beim MAR beschrieben, die von milden Formen bis hin zur follikulären Zerstörung des Haarfollikels reichen.^7^ Das zusätzliche Auftreten von Pusteln kann jedoch derzeit noch nicht erklärt werden. Wir vermuten, dass aufgrund der schweren Follikulitis eine neutrophile Chemotaxis ausgelöst werden könnte.

Es finden sich vermehrt Hinweise darauf, dass das Auftreten eines MAR mit verbesserter Ansprechrate einhergeht. Unser Patient zeigte ein schnelles und anhaltendes klinisches Ansprechen (partielle Remission). Die Behandlung des MAR richtet sich nach dem Schweregrad und der Beeinträchtigung der Lebensqualität. Empfohlen werden topische oder systemische Glukokortikosteroide, eine symptomatische Therapie mit antipruriginösen Medikamenten (zum Beispiel Antihistaminika) sowie – falls erforderlich – der zusätzliche Einsatz von Methotrexat.^11^ Ein Absetzen von Mogamulizumab sollte in Abhängigkeit vom Schweregrad des MAR individuell geprüft werden.^6^, ^11^ In dem hier beschriebenen Fall wurde die Therapie mit Mogamulizumab trotz des Auftretens eines MAR Grad III (> 30 % der Körperoberfläche) fortgeführt, da die Lebensqualität des Patienten nicht eingeschränkt war. Es handelt sich zudem um den ersten Bericht einer effektiven Behandlung eines MAR mit Doxycyclin.

Zusammenfassend zeigt der MAR ein breites Spektrum klinischer und histologischer Erscheinungsformen. Die Differenzierung gegenüber der Grunderkrankung gestaltet sich oftmals schwierig.^3^, ^6^ Aktuelle Studienergebnisse deuten darauf hin, dass das Auftreten eines MAR mit signifikant besserem Therapieansprechen einhergeht. Aus diesem Grund ist die Unterscheidung von einem Krankheitsprogress besonders wichtig, um ein verfrühtes Absetzen von Mogamulizumab zu vermeiden.^3^, ^6^

## DANKSAGUNG

Open access Veröffentlichung ermöglicht und organisiert durch Projekt DEAL.

## INTERESSENKONFLIKT

N.B. erhielt Honorare für Vorträge und Unterstützung für die Teilnahme an Tagungen und/oder Reisen von Kyowa Kirin. I.H.‐A. erhielt Unterstützung für die Teilnahme an Tagungen und/oder Reisen von Kyowa Kirin.

## References

[1] Kim YH , Bagot M , Pinter‐Brown L , et al. Mogamulizumab versus vorinostat in previously treated cutaneous T‐cell lymphoma (MAVORIC): an international, open‐label, randomised, controlled phase 3 trial. Lancet Oncol. 2018;19(9):1192‐1204. 30100375 10.1016/S1470-2045(18)30379-6

[2] Hirotsu KE , Neal TM , Khodadoust MS , et al. Clinical Characterization of Mogamulizumab‐Associated Rash During Treatment of Mycosis Fungoides or Sézary Syndrome. JAMA Dermatol. 2021;157(6):700‐707. 33881447 10.1001/jamadermatol.2021.0877 PMC8060888

[3] Avallone G , Roccuzzo G , Pileri A , et al. Clinicopathological definition, management and prognostic value of mogamulizumab‐associated rash and other cutaneous events: A systematic review. J Eur Acad Dermatol Venereol. 2024;38(9):1738‐1748. 38279614 10.1111/jdv.19801

[4] Pileri A , Clarizio G , Zengarini C , et al. Mogamulizumab‐associated rashes, their presentation and prognostic significance: a single‐centre retrospective case series analysis. J Eur Acad Dermatol Venereol. 2023;37(5):e615‐e617. 36545932 10.1111/jdv.18831

[5] Mitteldorf C , Langer N , Kempf W , Schon MP . Mogamulizumab‐associated rash simulating lupus miliaris disseminatus faciei. J Eur Acad Dermatol Venereol. 2023;37(4):e479‐e481. 36377614 10.1111/jdv.18741

[6] Hansen I , Abeck F , Menz A , et al. Mogamulizumab‐associated rash – Case series and review of the literature. J Dtsch Dermatol Ges. 2024;22(8):1079‐1086. 38924340 10.1111/ddg.15432

[7] Wang JY , Hirotsu KE , Neal TM , et al. Histopathologic Characterization of Mogamulizumab‐associated Rash. Am J Surg Pathol. 2020;44(12):1666‐1676. 32976123 10.1097/PAS.0000000000001587

[8] L'Orphelin JM . An occurrence of eosinophilic folliculitis and alopecia associated with a sustained complete response to mogamulizumab in Sezary syndrome: a case report. Ther Adv Hematol. 2024;15:20406207241235777. 38456078 10.1177/20406207241235777 PMC10919142

[9] Gorji M , Joseph J , Pavlakis N , Smith SD . Prevention and management of acneiform rash associated with EGFR inhibitor therapy: A systematic review and meta‐analysis. Asia Pac J Clin Oncol. 2022;18(6):526‐539. 35352492 10.1111/ajco.13740

[10] Bonnet P , Battistella M , Roelens M , et al. Association of autoimmunity and long‐term complete remission in patients with Sezary syndrome treated with mogamulizumab. Br J Dermatol. 2019;180(2):419‐420. 30328116 10.1111/bjd.17320

[11] Musiek ACM , Rieger KE , Bagot M , et al. Dermatologic Events Associated with the Anti‐CCR4 Antibody Mogamulizumab: Characterization and Management. Dermatol Ther (Heidelb). 2022;12(1):29‐40. 34816383 10.1007/s13555-021-00624-7 PMC8776934

